# *Salpa* genome and developmental transcriptome analyses reveal molecular flexibility enabling reproductive success in a rapidly changing environment

**DOI:** 10.1038/s41598-023-47429-6

**Published:** 2023-11-29

**Authors:** Kate R. Castellano, Paola Batta-Lona, Ann Bucklin, Rachel J. O’Neill

**Affiliations:** 1 Department of Molecular and Cell Biology, University of Connecticut, Storrs, CT USA; 2 Institute for Systems Genomics, University of Connecticut, Storrs, CT USA; 3 Department of Marine Sciences, University of Connecticut, Groton, CT USA; 4 Department of Genetics and Genome Science, University of Connecticut Health Center, Farmington, CT USA

**Keywords:** Marine biology, Computational biology and bioinformatics, Genetics

## Abstract

Ocean warming favors pelagic tunicates, such as salps, that exhibit increasingly frequent and rapid population blooms, impacting trophic dynamics and composition and human marine-dependent activities. Salp blooms are a result of their successful reproductive life history, alternating seasonally between asexual and sexual protogynous (i.e. sequential) hermaphroditic stages. While predicting future salp bloom frequency and intensity relies on an understanding of the transitions during the sexual stage from female through parturition and subsequent sex change to male, these transitions have not been explored at the molecular level. Here we report the development of the first complete genome of *S. thompsoni* and the North Atlantic sister species *S. aspera.* Genome and comparative analyses reveal an abundance of repeats and G-quadruplex (G4) motifs, a highly stable secondary structure, distributed throughout both salp genomes, a feature shared with other tunicates that perform alternating sexual-asexual reproductive strategies. Transcriptional analyses across sexual reproductive stages for *S. thompsoni* revealed genes associated with male sex differentiation and spermatogenesis are expressed as early as birth and before parturition, inconsistent with previous descriptions of sequential sexual differentiation in salps. Our findings suggest salp are poised for reproductive success at birth, increasing the potential for bloom formation as ocean temperatures rise.

## Introduction

**Figure 1:**
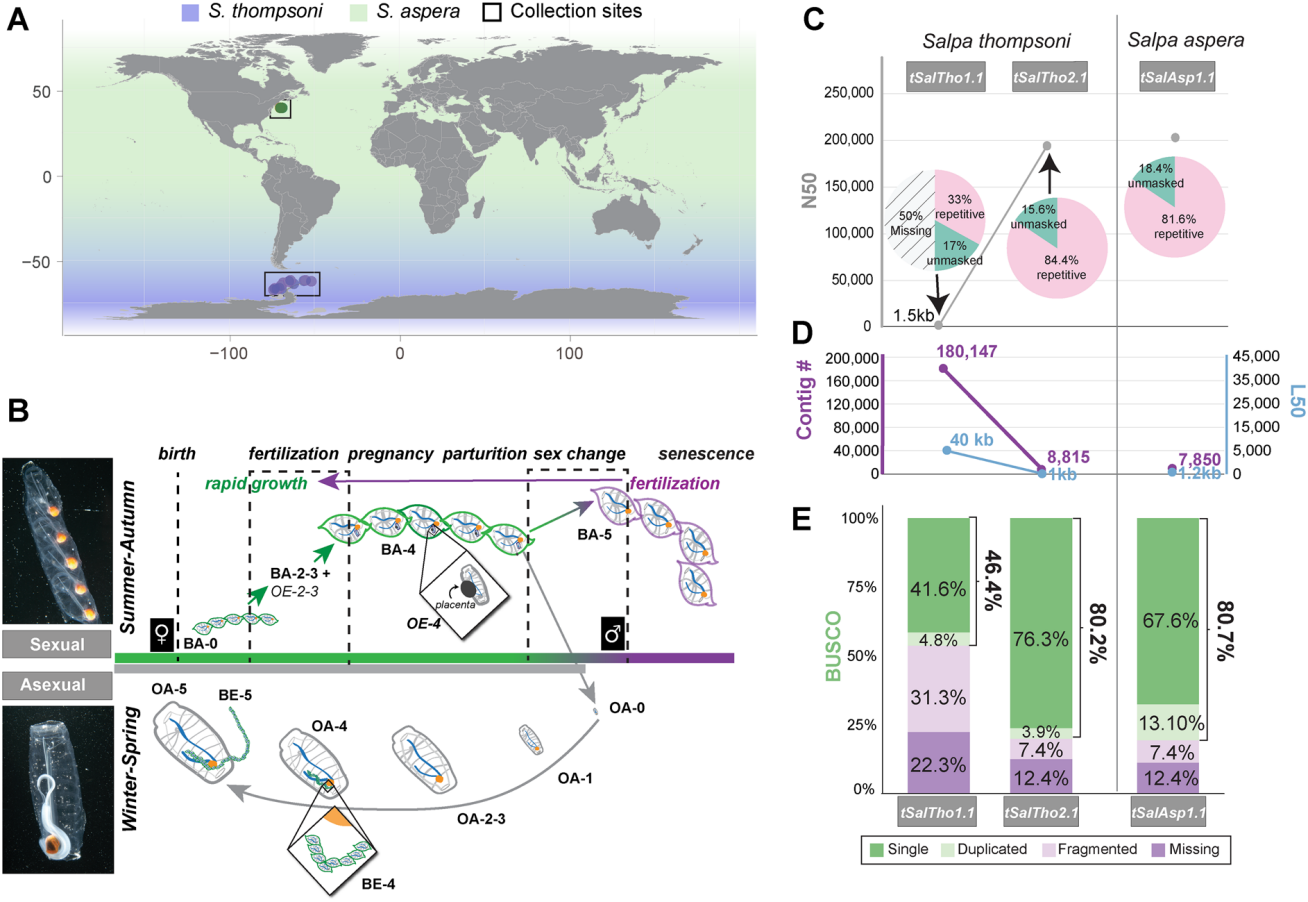
Habitat, life cycle and genome assemblies for *S. thompsoni* and *S. aspera.* (**A**) *S. thompsoni* resides in the Southern Ocean (purple shading) while *S. aspera* is found broadly around the globe (green shading). Samples used for this study are represented by purple (*S. thompsoni*) and green circles (*S. aspera*) according to longitude and latitude at the site of collection. The map was generated by plotting latitude and longitude onto a world base map with *ggplot2* v3.3.5^108^ in *RStudio* v2022.07.1 + 554^109^. (**B**) The reproductive life cycle of salps alternates seasonally between aggregate blastozooids (top left) which perform sexual reproduction during the summer-autumn seasons and solitary oozooids (bottom left) which perform asexual reproduction during the winter-spring. Blastozooids have been described as protogynous hermaphrodites that transition from female (green salps) at birth (BA-0) to male (purple salps) (BA-5) after parturition. Males mate with early-stage females (BA-2, BA-3), purple arrow, prior to senescence. Each female (~ BA-4) gives birth to a solitary oozooid (grey salps) (OA-0) that matures over winter in deep waters, asexually producing an aggregate chain of blastozooid embryos (BE) that are released in summer. Zoom insets show the OE and associated placenta (top) and BA chain. Stages: Blastozooid adult (BA), Oozooid Embryo (OE), Oozooid adult (OA), Blastozooid Embryo (BE), # is the developmental stage number^20^. Photos by Larry Madin. (**C**) Comparison of genome completeness of the original draft assembly *tSalTho1.1* and *tSalTho2.1* and *tSalAsp1.1* assemblies. Pie charts represent the entirety of the expected genome size, missing genome portions shown with black lines, colored slices represent the percentage of the assembly masked/unmasked by repeats. The new assemblies show an overall two-fold improvement in assembly completeness and contiguity, represented by increased N50 values (**C**), low contig and L50 values (**D**), and improved *BUSCO* completeness scores (**E**).

Salps are a key group of gelatinous chordates (Tunicata, Thaliacea) that exhibit rapid shifts in population density and distribution, known as blooms, that result in dramatic impacts on ocean ecosystems, including food webs^1, 2^ and carbon flux^3–6^ with concomitant impacts on international fisheries^7^ and energy resources such as nuclear power plants^8, 9^. Encompassing 12 genera and an estimated 72 known species, salps are found globally. Two species, *Salpa thompsoni* and *S. aspera,* dominate the Southern and Northwest Atlantic Ocean, respectively (Fig. 1A). Both regions are experiencing marked shifts in climate^10–12^ with documented evidence of increasing density and frequency of salp blooms^1, 2, 13, 14^ that cover large areas (up to 100,000 km^2^)^13^ and comprise up to 99% of the biomass of the zooplankton community^15^. Salp bloom formation, combined with highly efficient filter feeding^16^, significantly reduces primary production and availability of phytoplankton for other consumers and results in radical shifts in the distribution and abundance of essential trophic species with devastating impacts on food web dynamics^1, 2, 17^ and human activities^8, 9^.

Salp blastozooid chains can quickly form large swarms, known as blooms, during the summer months as they grow rapidly (10–20% in length per hour)^18^, coincident with the sexual stage of reproduction. The salp reproductive strategy consists of seasonal alternations between an asexual solitary (oozooid) form that overwinters and sexual aggregate (blastozooid, forming chains) life form that are considered protogynous (or sequential) hermaphrodites with testis maturation occurring after parturition (Fig. 1B)^14^. Recent modeling showed that a “proportional growth rate hypothesis” best fit observed *S. thompsoni* population dynamics, wherein reproduction rate, influenced by the number of oozooids that produce chains in the spring, coupled with favorable conditions during and following embryo release, was the critical factor influencing population levels^19^. However, our understanding of salp reproduction is limited to visual descriptions^14, 20, 21^, inherently difficult due to the fragility of *Salpa* bodies, transparent tissue and the inability to maintain individuals in a laboratory long-term. Despite the ecological and economic devastation of increasing salp blooms, the genetic mechanisms regulating their growth and reproductive life history are still unknown, rendering predictions of bloom formations and their ecological and economic impact challenging and incomplete.

To support a genomics-based approach for understanding salp blooms and reproductive strategies, we generated long-read based genome assemblies for *S. thompsoni* and *S. aspera,* providing the only two reference genomes for salp species. The new *S. thompsoni* assembly vastly improves upon the previously published draft assembly^22^ and fills in missing genomic data. Distinguishing salps from other tunicates, we find that high repeat content typifies salp genomes, with > 80% of total genome content annotated as repetitive. In addition, unique sequencing signatures from nanopores (Oxford Nanopore Technologies) and computational predictions led to the identification of a high density of G-quadruplex motifs enriched near genes, suggesting a role for secondary structures in transcriptional regulation. Transcriptomics and miRNA analyses across developmental stages for both blastozooid and oozooid salps revealed that testis maturation and fertilization/embryo development occur simultaneously and at earlier stages of the blastozooid life history than previously known. Our findings, coincident with increases in rapid growth rates during early sexual reproduction, suggest salps are poised for sperm release as soon as the environmental stimuli are available, increasing the potential for blooms during warming trends affecting our current bloom predictions.

## Results and discussion

### Building genomic tools for *S. thompsoni* and *S. aspera*

The *S. thompsoni* genome was sequenced with Oxford Nanopore Technologies (ONT), yielding 188 × coverage, based on the previously estimated genome size of 602 Mb (± 173 Mb))^22^ (Supplemental Table S1). Comparisons among assembly algorithms and polishing methods were performed and the highest quality assembly was selected based on N50, L50 and Benchmarking Universal Single-Copy Ortholog (BUSCO)^23^ metrics (Supplemental Table S2). A de novo assembly using Shasta^24^ produced a 742 Mb final assembly (*tSalTho2.1)* consisting of 8,815 contigs with a N50 = 191 kb and L50 = 1,071 (Fig. 1C,D), and BUSCO = 80.2% complete, with 3.9% duplicated and 7.4% fragmented (Fig. 1E). *tSalTho2.1* represents a significant improvement over the *S. thompsoni* 1.0 draft assembly (*tSalTho1.1),*^22^ which contained only half the expected genome size (318 Mb) in 180,147 contigs with a N50 = 1.5 kb and L50 = 40,059 (Fig. 1C,D) and BUSCO = 46.4% (Fig. 1E). To confirm high genome assembly integrity, raw DNA reads from independent sequencing runs were mapped to the assembly with high accuracy (99.15–99.62%) (Supplemental Table S3). While *tSalTho2.1* was a significant improvement over *tSalTho1.1,* further scaffolding using Dovetail Omni-C proximity ligation proved unsuccessful due to the high number of contigs and exceptionally high repeat content (Supplemental Table S4). It is important to note that our analyses resulted in low assembly contiguity, despite the acquisition of high molecular weight DNA (Supplemental Fig. S1A), due to low sequencing efficiency (defined as shorter than expected read length (Supplemental Fig. S1B)), average of 2.5 kb from five flow cells (Supplemental Table S1), and fewer than expected reads per flow cell, 161 GB from five total flow cells (Supplemental Fig. S1C,D). A test utilizing whole genome amplification (WGA) mitigated low sequencing efficiency (Supplemental Fig. S1E,F) suggesting a secondary structure was inhibiting nanopore sequencing (see *G-quadruplex motifs are abundant in salp genomes*). However genome coverage comparisons between ONT 1D and WGA runs showed no large change in coverage genome wide (Supplemental Fig. S1G) and despite the increased number of reads, read coverage was the same for both the ONT 1D and WGA runs (50% of the genome had ~ 30% coverage) (Supplemental Fig. S1G). While informative about the possibility of secondary structures, WGA reads resulted in reduced assembly contiguity and were therefore excluded from the final assembly to avoid introducing assembly errors (Supplemental Table S2).

Employing this same sequencing and assembly approach, we generated 283 × coverage of Oxford Nanopore long reads (Supplemental Table S1) and a final 903 Mb de novo assembly for *S. aspera (tSalAsp1.1)* using Flye^25^ (Supplemental Table S5). *tSalAsp1.1* consists of 7,850 contigs, with N50 = 202 kb and L50 = 1,215 (Fig. 1C,D). Additionally, *tSalAsp1.1* had a BUSCO score of 80% complete, with only 13% duplicated and 8.10% fragmented (Fig. 1E), and raw read mappability of 99.15–99.62%, on par with *tSalTho2.1* (Supplemental Table S3). The genome size difference between *S. thompsoni* and *S. aspera* may be influenced by a higher heterozygosity in *S. aspera,* making it difficult to determine and remove haplotigs from our assembly and possibly contributing to the 4 × higher BUSCO duplication rate of 13%. As observed for *S. thompsoni,* genome scaffolding using Dovetail Omni-C was unsuccessful for *S. aspera* due to the high contig number and high repeat content (Supplemental Table S4). Taken together, the low mappability rates for Omni-C libraries for each species are strikingly similar (*S. thompsoni* 21.12% and *S. aspera* 15.59%), suggesting the inherently high repeat content of the salp genomes precludes scaffolding using short sequencing reads.

Previously, a whole-transcriptome analysis comparing seasonal (austral spring and summer 2011) and geographical (off-shelf, on-shelf, Bransfield Strait) transcriptional shifts in *S. thompsoni* in the Western Antarctic Peninsula (WAP) identified differentially expressed genes associated with both environmental stress and reproduction^26^. However, this transcriptome prioritized adult blastozooid stages and therefore did not allow inferences about reproductive mechanisms. To better understand the reproductive mechanisms required to support alternating reproductive life histories and expansive bloom formation, we derived a pan-developmental transcriptome including 21 samples from blastozooid and oozooid adult and embryonic stages (Supplemental Fig. S2A, Supplemental Table S6). During dissection, the embryonic stages were removed from adult tissue and snap-frozen separately. The final de novo transcriptome contains 51,974 sequences ranging in size from 300 to 15,495 bp, an average length of 926.74 bp and a N50 length of 1,325 bp. Completeness of this transcriptome, as measured by BUSCO score, is 97.3%, slightly higher than the published environmental transcriptome at 94.9% (Supplemental Fig. S2B, Supplemental Table S7). The transcripts were annotated using *EnTAP*^27^; 54.7% of transcripts were annotated by gene family and/or similarity search (Supplemental Fig. S2C), while 45.3% remained unannotated. RNA-seq reads from each sample in the environmental^26^ and the developmental transcriptome (herein) map to *tSalTho2.1* at ~ 88% (Supplemental Table S8), while the merged developmental transcriptome maps at 75.65%. The high BUSCO score for the transcriptome (97.3%) suggests the transcriptome is near complete, facilitating comprehensive gene expression comparisons among salp stages. The discordance between the transcriptome BUSCO score and genome mapping rate of RNA-seq reads suggests some genes may still be missing from our genome assembly. In addition, the low annotation rate (54.7%), while unsurprising for a non-model genome, will render identification of some novel pathways difficult.

### G-quadruplex motifs are abundant in salp genomes

One major challenge to deriving fully contiguous assemblies for salp genomes is the low sequencing efficiency obtained during ONT sequencing. The increase in sequencing efficiency and yield from Whole Genome Amplification (WGA) coupled with no increase in genome coverage (Supplemental Fig. S1F,G) suggested that there may be a non-B-form secondary structure preventing adapter ligation to the DNA molecules, reducing efficient sequencing through the nanopore (Supplemental Fig. S1G). One such structure that was tested are G-quadruplexes (G4), stable secondary structures that form in guanine rich regions of both DNA and RNA^28, 29^. G4s were selected based on the high repeat content of the salp genome and the ability to computationally predict G4 sequence motifs.

**Figure 2:**
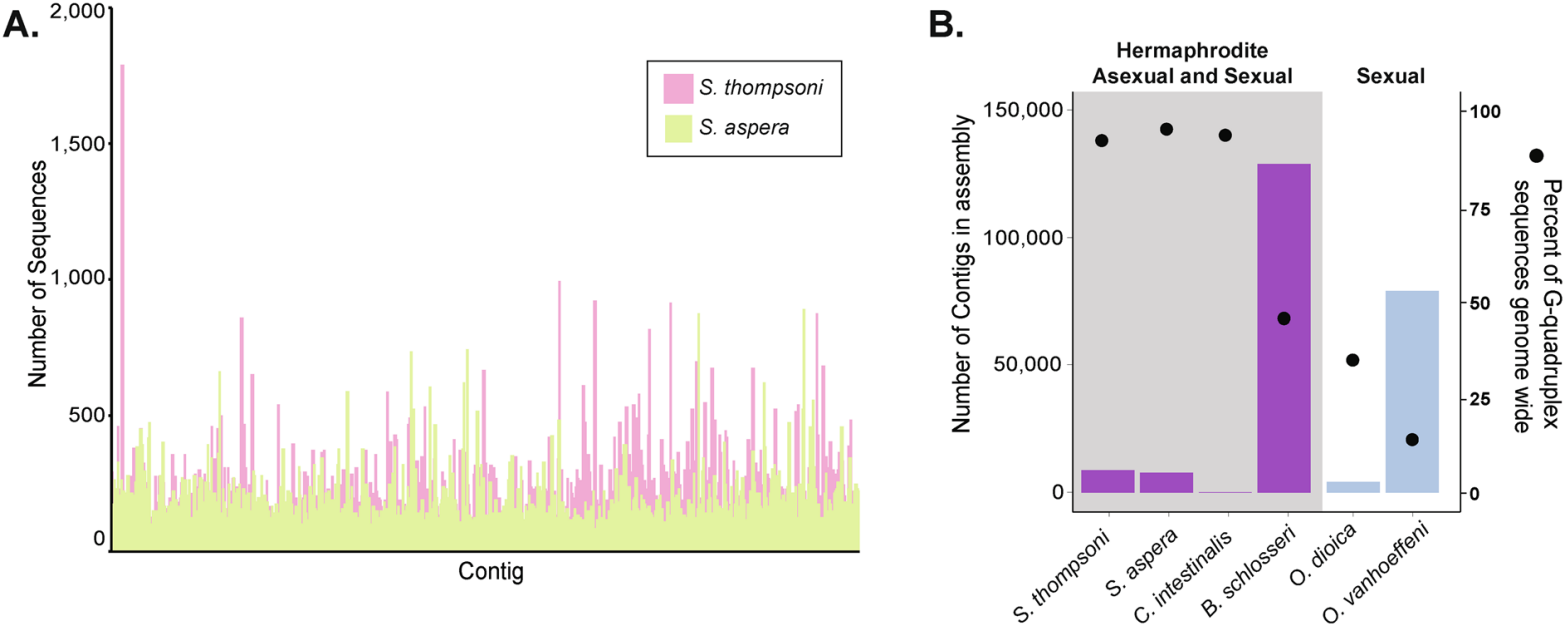
G-quadruplex (G4) motifs are abundant in salp genomes and other tunicate species with alternating sexual and asexual reproductive modes. (**A**) G4 motifs are abundant throughout the genomes of *S. thompsoni* (pink) and *S. aspera* (green). Plot shows density of G4 sequences for each contig comprising each genome assembly. (**B**) Comparison of genome percentage of G4 motifs (black dot) among tunicate species shows they are abundant in tunicates with both sexual and asexual reproductive life cycles (grey box and purple bars) versus those that solely perform sexual reproduction (blue bars), independent of genome assembly fragmentation. Bars represent the number of contigs in the assembly.

Through computational predictions of G4 motifs, G4s are found to be abundant throughout both the *S. thompsoni* and *S. aspera* genomes. The *S. thompsoni* genome contains 198,942 total G4 motifs on 93% of contigs and *S. aspera* contains 398,110 total G4 motifs on 94% of contigs (Fig. 2A, Supplemental Table S9). When compared to other tunicates, the total number of G4-motifs is 2–75 × and 4–135 × more abundant in *S. thompsoni* and *S.aspera* assemblies, respectively (Supplemental Table S9). Interestingly, G4 motifs were found to be the most abundant (55–110 × fold difference) in species with hermaphroditic and/or alternating reproductive life histories (*S. thompsoni, S. aspera, B. schlosseri* and *C. intestinalis*) versus those that only perform sexual reproduction with defined sexes (*O. dioica* and *O. vanhoeffeni*) (Fig. 2B)*.* In addition, G4 motifs are not equally distributed in the genome, with *S. thompsoni* carrying 0–842 G4 motifs per 10 kb (equal distribution expectation: 5 motifs per 10 kb) with as many as 19.6% of 10 kb regions containing no G4 motifs, and *S. aspera* carrying 0–231 G4 motifs per 10 kb (equal distribution expectation: 14 motifs per 10 kb) with 13% of fragments containing no G4s (Supplemental Table S9). The difference in G4 motif abundance and unequal distribution throughout the genomes suggests they may be clustering around certain regions such as genes or repeats. In *S. thompsoni*, G4 motifs are found in predicted cis-regulatory regions (CREs, regions within 5 kb upstream of the start) of 61.8% of predicted genes (Supplemental Table S10). Interestingly, genes with G4 motifs in predicted CREs include those involved in sex determination and cell proliferation control: *Sox10, Sox30, FoxL2, FoxA2, FOXC2, RBFOX2, Doublesex* and *mab-3 related transcription factors* (*DMRT3* and *DMRT2A*). It is unclear if G4 motifs are forming secondary structures or how they are functioning around genes; however, finding G4 motifs around many genes, particularly those involved in sex determination, suggests they may be important regulators of gene expression.

### The *S. thompsoni* and *S. aspera* genomes are composed of > 80% repetitive sequences

**Figure 3:**
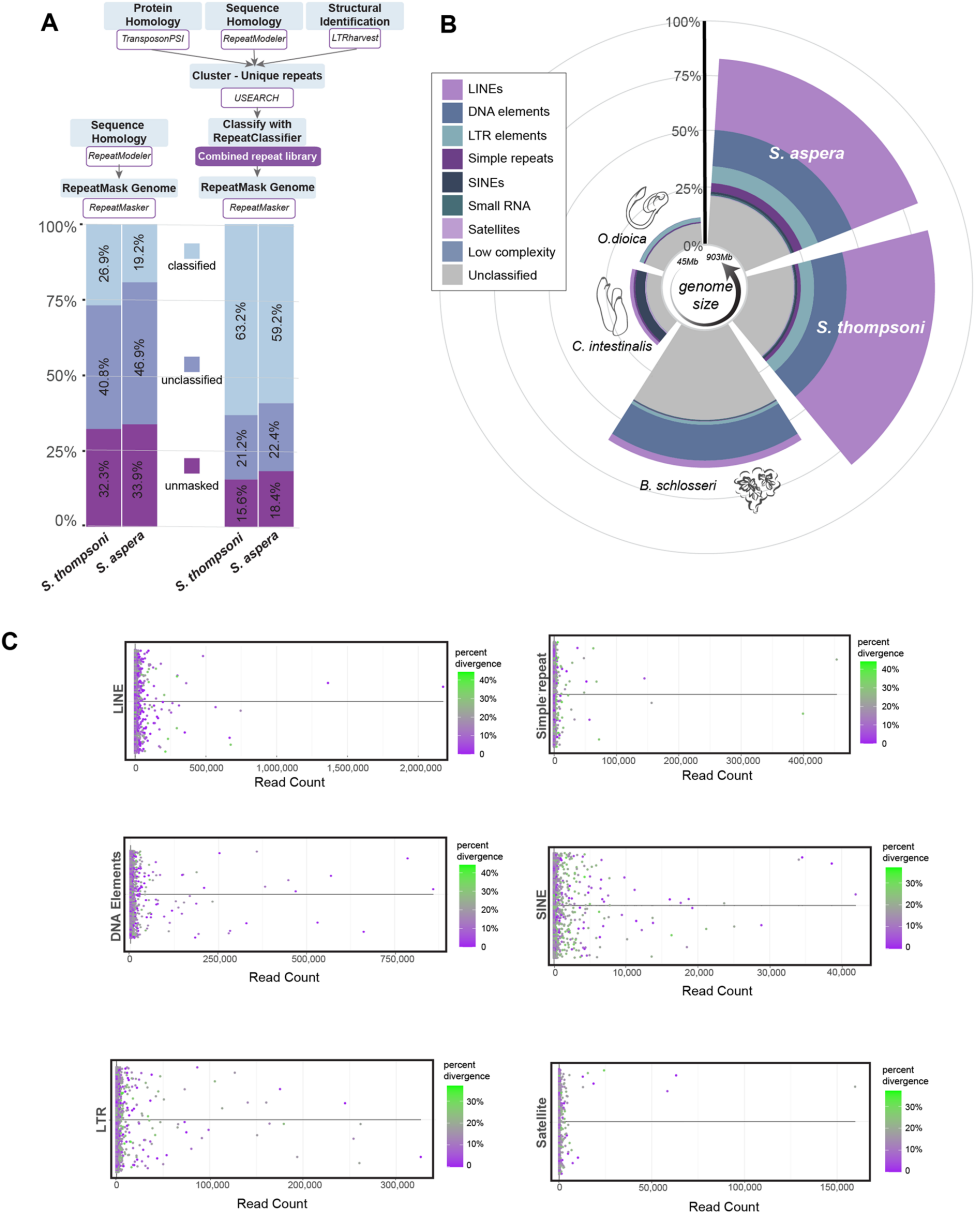
*S. thompsoni* and *S. aspera* genomes carry high repeat content. (**A**) Comparison of the traditional repeat annotation pipeline (left) to the combined approach employed herein (right). The combined approach yields a higher percentage of classified repeats (light blue) compared to unclassified (dark blue) and unmasked (purple). (**B**) Comparison of the genome percentage annotated as repetitive, with a breakdown of the proportion of repeat families (as per key to left) among tunicates with small (*O. dioica*) to large (*Salpa*) genome sizes (scale in circle inset). (**C**) Each box plot shows the number of RNA-seq reads for repeats in each repeat family in the *S. thompsoni* transcriptome. The percent divergence refers to the sequence similarity of each locus to the *RepeatModeler* consensus sequences. Higher divergence (green) indicates the repeat is older while low divergence (purple) indicates the repeat is young^33^.

Repeat annotation using a de novo* RepeatModeler* library and *RepeatMasker* (Fig. 3A) revealed that the *tSalTho2.1* genome is 67% repetitive, supporting previous predictions^22^, and the *S. aspera* genome is 66.14% repetitive. However, only 26.23% and 19.23% respectively of repeat elements could be classified with this method (Fig. 3A). Therefore, to reduce the percentage of unclassified repeats, a combined de novo repeat library was used merging three approaches: (1) traditional sequence homology (*RepeatModeler*) described above, (2) structural identification of known features including length, distance and sequence motifs (*LTRharvest*), and (3) protein homology (*TransposonPSI*). Using the combined de novo repeat library, more repeats were annotated revealing both salp genomes are ~ 80% repetitive, representing an increase of ~ 20% beyond previously predicted repetitive content (Fig. 3A, Supplemental Table S11)^22^. The combined de novo repeat library approach also increased the proportion of classified repeats from 40.8% to 63.2% in *tSalTho2.1* and 19.2% to 59.2% in *tSalAsp1.1*, increasing the overall percentage of classified repeats (Fig. 3A, Supplemental Table S11). Of the repeats annotated in salp, the most abundant classes are LINEs, DNA elements and LTR elements (highest to lowest) in both *S. thompsoni* and *S. aspera* (Fig. 3B, Supplemental Table S11)*.* All three element classes are found randomly distributed throughout both genomes (Supplemental Fig. S3).

Genome size differences as large as 12 × have been observed between tunicate species and previous repeat analyses revealed that genome size correlates with transposable element (TE) abundance^30^. For example, *B. schlosseri* has the largest genome published thus far at 742 Mb and 66% repeat content while *O. dioica* has the smallest genome*,* 70 Mb, and 15% repeat content^31, 32^(Fig. 3B). *S. thompsoni* and *S. aspera* follow this trend, exhibiting larger genome sizes (742 Mb and 901 Mb, respectively) and higher repeat content (84% and 81%, respectively) than other tunicates (Fig. 3B). In addition, the abundant repeat classes in salps (LINEs, DNA elements and LTR elements) differ from other tunicates; LTR elements are most abundant in both *Oikopleura* species, SINEs are the most abundant class seen in *C. intestinalis*, and DNA elements are most abundant in *B. schlosseri* (Fig. 3B)*.* The most abundant LINE elements in salp, RTE-BovB and CR1, have not been identified in *C. intestinalis* or *O. dioica*. On the other hand, the gypsy LTR elements are shared among all tunicates analyzed, including species with the smallest tunicate genome to date, *O. dioica*^30^ (Supplemental Table S12). The addition of the *S. thompsoni* and *S. aspera* genomes in this repeat comparison confirms and accentuates the large variation in repeat family abundance across tunicates with differing genome sizes, suggesting there is variability in repeat activity that has contributed to genome size changes observed among tunicate species.

Utilizing the *S. thompsoni* pan-development transcriptome, repeat transcription and repeat divergence from the RepeatMasker consensus were analyzed. Repeats with low divergence and high transcription are considered evolutionarily young and potentially active elements while those with high divergence (more mutations) and low (or no) transcription are considered older, inactive elements^33^. Overall, known mobile element families that are most abundant in the genome (LINEs, DNA elements (inclusive of DNA transposable elements and helitrons) and LTRs) also have the highest transcription levels (Fig. 3C), indicating the activity of young elements contributed to genome size expansion. Transcripts are produced by many repeat sequences in the genome, indicating that there are many different young, active elements that may have contributed to genome size and TE diversity rather than a burst of activity from a single element type (Fig. 3C). While most repeats are categorized as immobile (high divergence and low or no transcriptional signal), many repeats in the top represented repeat families show low divergence (< 10%) and high transcription suggesting they may still retain transpositional activity in the *S. thompsoni* genome (Fig. 3C). Many repeats that remained unclassified (“unknown” repeats) are found in the *S. thompsoni* transcriptome and show high transcriptional activity suggesting these are repeats of recent origin specific to salps. Repeat subfamilies with low divergence and high transcription in the LINE family include I, RTE-BOVB, and L2s (Fig. 3C). LINE-L1s, a subfamily shown to still be active in the human genome^34, 35^, show some transcriptional activity in salp genomes, are represented by copies with 0% divergence and are likely full length (3500–9400 kb). However, a majority of the transcriptionally active L1s carry higher sequence divergence (10–30%) and are truncated, suggesting they likely are no longer capable of retrotransposition. The most transcriptionally active DNA elements are the hATs. While SINEs comprise only 0.89% of repeats in the *S. thompsoni* genome, unknown SINE and *Alu*-like elements show transcriptional activity. The mean sequence divergence of the *Alu-*like elements is 14.8% and length is 248 bp. The observation of transcriptionally active repeats in the *S. thompsoni* genome supports previous hypotheses^30, 36^ that the large genomic changes and rapid evolution observed among tunicates are due to repeat invasion, mobility, and increased TE copy number.

### Low gene density is reflective of genome expansion in *S. thompsoni*

A final total of 26,610 gene models were predicted for *S. thompsoni* by *Braker*^37–41^ with a BUSCO completeness score of 65.30%, 2.4% duplicated and 14.60% fragmented (Supplemental Table S13). To obtain this final set, gene models from *Braker* were filtered to remove erroneous gene models and resolve conflicting annotations to refine gene models. Gene models were checked with *BUSCO* to ensure universal single copy orthologs were not lost after filtering steps and to determine the best filtering options for this set of genes (Methods, Supplemental Fig. S4). Of the predicted genes, 20,907 have introns and 5633 are single exon genes. The average gene length is 7.59 kb with a mean exon length of 160 bp and an average number of six exons per multi-exonic gene (Supplemental Table S13). Of the 26,610 genes, *EnTAP*^27^ annotated 19,253 by gene family and/or similarity search, while 7357 remain unannotated. Of the annotated genes, the top ontology categories include system development, cell differentiation, animal organ development and embryo development (Supplemental Table S13).

Gene density among tunicates varies with genome size, with lower gene density in larger genomes and higher genome density in tunicates that have undergone genome compaction^36^. *C. intestinalis* and *O. dioica* have undergone different levels of genome compaction and have 7.6 × and 10 × smaller genomes, respectively, than *S. thompsoni*^36^. In *S. thompsoni,* gene density is observed at 1 gene per 27.9 kb, 2 × less dense than *C. intestinalis* and 7 × less dense than *O. dioica*. *S. thompsoni* gene density is also 1.8 × less dense than *B. schlosseri* which is the closest in genome size (580 Mb) and repeat abundance (65%)^31^. The low gene density supports previous hypotheses^36^ and suggests either (1) genome reductions have not occurred or have occurred very minimally, or (2) genome reductions have occurred but were countered by expansions of repeats.

### Novel stage-specific small RNAs in the alternating reproductive life history of *S. thompsoni*

**Figure 4:**
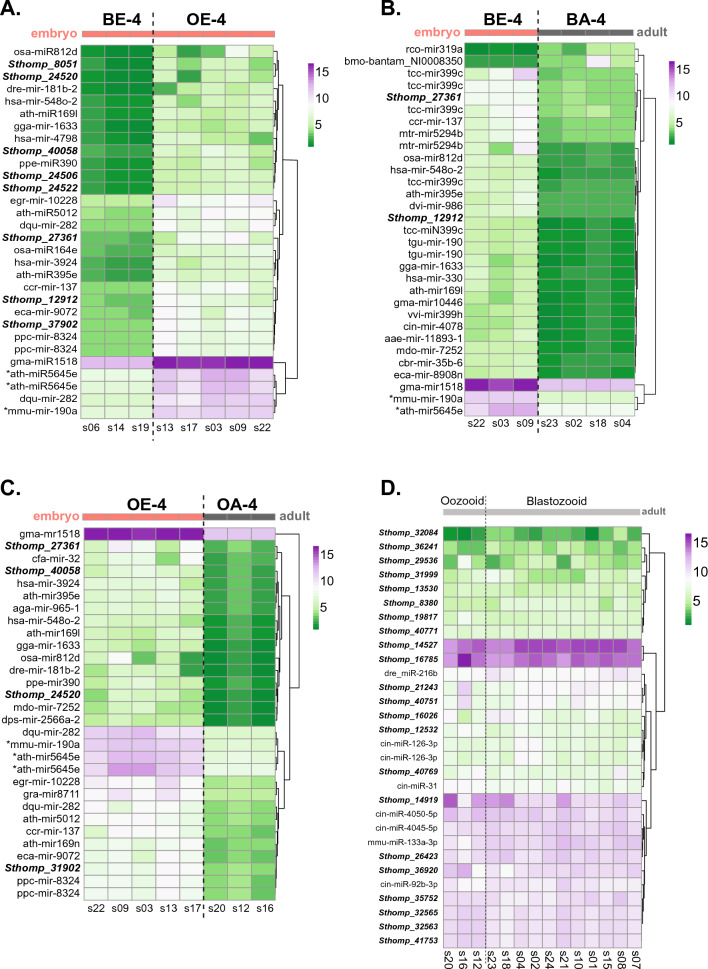
(**A**–**C**) Heatmap of top 30 differentially expressed miRNAs among all embryonic stages (**A**), blastozooid embryos (BE) and blastozooid adults (BA) (**B**), and oozooid embryos (OE) and oozooid adults (OA) (**C**). (**D**) Heatmap of top 30 differentially expressed miRNAs among all adult oozoid and blastozoid samples. The color scale represents the log2 fold change calculated from DEseq2. Left of each heat map: known miRNA annotations are indicated while miRNAs with no orthology to known miRNAs are italicized/bold and labeled *Sthomp_#.* Asterisks denote miRNAs upregulated and shared across all three comparisons (**A**–**C**)*.* Embryo stages are denoted by orange bar at top of graph; adult stages are denoted by grey bar at top of graphs.

Due to the prominent role of microRNAs (miRNAs) in development and cell proliferation^42–44^, small RNA sequencing of developmental stages (the same samples and stages used for mRNA sequencing) was completed to identify miRNA profiles that distinguish sexual and asexual reproductive phases. In tunicates, the only known sex-specific miRNAs (miR1478, 1487 and 1488) have been identified in *O. diocia*^45^*,* a solely sexually reproducing species. miR1478, 1487 and 1488 were not identified in *S. thompsoni*. When comparing embryonic stages, five miRNAs were upregulated in the oozooid (OE-4) embryos versus blastozooid embryos (BE-4) suggesting different miRNAs and miRNA mechanisms are required for regulating asexual versus hermaphroditic reproduction (Fig. 4A, purple). In addition, two of those same miRNAs (ath-miR5645e and mmu-mir-190a) were upregulated in blastozooid embryos (BE-4) and oozooid (OE-4) embryos when compared to their respective blasto- and oozooid adults (Fig. 4B,C). While many of the miRNAs in the top 30 of those differentially expressed were annotated, more unannotated differentially expressed miRNAs (denoted by Sthomp_#) were observed in the embryo (OE vs BE) comparisons (Fig. 4A–C). In contrast to embryo comparisons, the top miRNAs expressed among oozooid and blastozooid adult stages are shared (Fig. 4D). While these data show that salp reproductive cycles are defined by differentially expressed suites of miRNAs, prediction of their responsive targets requires consideration of efficiency of canonical and non-canonical binding sites in the miRNA seed as well as potential target mRNA expression levels that are impacted by concomitant miRNAexpression^46^. Thus, further investigation into their biogenesis, target genes and tissue localization utilizing functional target assays would help decipher if they have a specific function during salp reproduction or other stage-specific processes.

### Differential gene expression suggests blastozooid salp perform spermatogenesis prior to parturition

**Figure 5:**
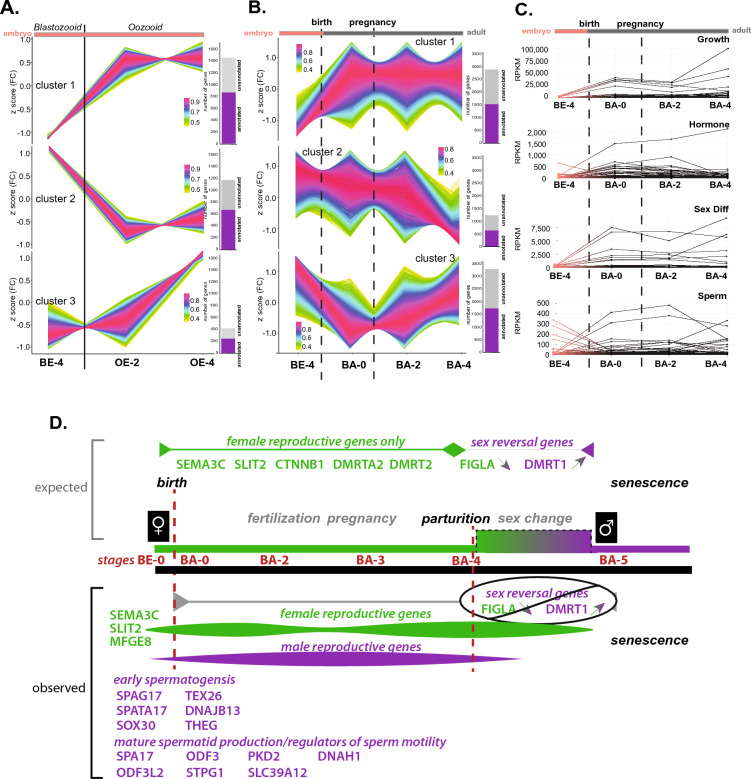
Multi-stage and cross-lifecycle transcriptomes reveal convergence of growth, sex differentiation and sperm production following birth. Clusters of differentially expressed (DE) genes among blastozooid embryos (BE) and oozooid embryos (OE) (**A**) and among all blastozooid stages (BE-4 through BA-4) (**B**). From top: clusters 1–3. Normalized read counts were transformed using z-score transformation to reduce noise introduced by absolute values. The color scale represents the degree to which each gene belongs to the cluster with pink representing the highest membership and yellow representing the lowest. To the right of each graph are the proportion of annotated (purple) and unannotated (grey) genes in the total gene pool for each cluster. Vertical line in (**A**) separates BEs from OEs and in (**B**) indicates the timing of birth and pregnancy. (**C**) RPKM for genes annotated by category (from top: growth, hormone, sex differentiation, and sperm production) in the BE-4 embryo stage, prior to birth (first vertical dotted line from left), and adult stages between birth and pregnancy (second vertical dotted line from left), BA-0, and after parturition, BA-2 and BA-4. (**D**) Expected (top) and observed (bottom) gene expression profiles across the blastozooid life stages. Green demarcates female expression, purple demarcates male expression. The timing of specific events are noted (birth and parturition) with vertical dotted lines. In the expected model, salps are born female, with presumed functioning female gonads only. Just prior and following parturition, salps undergo a sex change.

While physical descriptions of salps and their reproduction exist^14, 20^, no molecular analyses of salp developmental stages have been completed to date, severely limiting our understanding of salp reproduction and its influence on bloom frequency and density. To identify genes that represent and regulate the complex life history of salp species, differential expression and temporal clustering among stages were explored (Fig. 5, Supplemental Figs. S5, S6). In order to identify the genes involved in the alternate reproductive forms, sexually and asexually produced embryos were compared. Initial comparisons of embryonic stages (blastozooid embryo stage 4 (BE-4), oozooid embryo stage 2 (OE-2), and oozooid embryo stage 4 (OE-4)) revealed tight clustering of genes with expression changes that differentiate blastozooid (BE-4) and oozooid (OE-2 and -4) embryo stages (Fig. 5A, clusters 1–3). Enriched GO terms in cluster 1 include reproductive behavior and regulation of growth (Supplemental Fig. S6A, Supplemental Table S14) and those in cluster 2 describe processes such as aging, regulation of reproductive process, multicellular process and growth, mesenchymal cell proliferation and growth factor binding (Supplemental Fig. S6B, Supplemental Table S15). Enriched GO terms in cluster 3 include fertilization, gamete generation, regulation of growth and regulation of developmental processes, all of which are expected as fertilization and embryo development of the asexual buds is known to occur from OE-2 until birth (Supplemental Fig. S6C, Supplemental Table S16). However, looking closely at all GO terms in cluster 1 revealed the expression of genes involved in sex differentiation in BE-4, a process which was not thought to occur until after release of a developed embryo at BA-4 (Supplemental Fig. S5A, Supplemental Tables S17–S20).

During blastozooid reproduction, it was thought that there is a small period of sex change from female to male at stage BA-4, after the female has released the embryo (parturition). These males are then available to fertilize earlier stage (BA-2) salps^14, 21^. Based on previous studies of other sequential hermaphroditic marine species, it is predicted that protogynous hermaphrodites, such as salps, will carry skewed ratios of transcripts in the gonads, with only female-specific gene expression profiles observed prior to sex change (i.e. parturition in salps)^47, 48^. Just prior to parturition, gene expression profiles associated with the change in sex from female to male should be observed as the protogynous hermaphrodite starts to undergo sex change to male and subsequent fully functioning male gametes. To assess the timing and molecular triggers of sex change, differential expression was performed among available blastozooid stages (BE-4, BA-0, BA-2 and BA-4) (Figs. 1B and 5B,C, Supplemental Tables S21–S28). Herein we observe that the transition from BE to BA marks an increase in hormone production concomitant with growth, indicative of the initiation of the rapid growth salps undergo following birth^49, 50^ (Fig. 5C, Supplemental Tables S25–S26).

Contrary to predictions, however, the BE to BA transition and subsequent BA stages show expression of genes involved in both female and male sexual differentiation and mature sperm formation, with genes associated with sperm and sexual differentiation expressed as early as the adult blastozooid stage 0 (BA-0) (Fig. 5B–D, Supplemental Tables S27–S29). Moreover, genes involved in early spermatogenesis (e.g. *Sox30*^51, 52^*, DNAJB13*^53^*, SPAG17*^54^, *SPATA17*^55, 56^*, TEX26*^57^ and *THEG*^58, 59^) are expressed in the blastozooid embryo (BE-4), before birth, through stage BA-4. Interestingly, two of these genes, *SOX30*, an *SRY*-box transcription factor and key regulator of spermatogenesis, required for fertility in male mice^51, 52^ and *DNAJB13*, whose protein participates in spermatogenesis and aids in sperm motility, show strong male-specific expression patterns in protandrous hermaphroditic clownfish^53^, prior to changing to female. In all adult blastozooid stages, we find expression of genes involved in mature spermatid production (e.g.i.e. *SPA17, ODF3, ODF3L2, STPG1*) and genes that serve as key regulators of sperm motility and capacitation (e.g. *PKD2, DNAH1*, (reviewed in^60^)and *SLC39A12*^61^). Thus, salps may initiate spermatogenesis in blastozooid embryos prior to release and spermiogenesis in blastozooid adults prior to pregnancy, earlier than post-parturition, as had been previously described^14, 19, 20^.

In addition to male bias genes, female specific gene expression is also observed overlapping with male gene expression (Fig. 5D, Supplemental Tables S27, S29). Semaphorin-3C (*SEMA3C*) and Slit Guidance Ligand 2 (*SLIT2*) are required for follicle formation in mammal ovaries^62, 63^ and both show strong female biased expression in clownfish after sex change^53^. In salp, *SEMA3C* and *SLIT2* expression is highest in the blastozooid embryo (BE-4) but are expressed through BA-4. Milk fat globule epidermal growth factor 8 (*MFGE8*) has been found to be significantly upregulated during the window of implantation in humans^64, 65^. In salp, *MFGE8* first shows expression in BA-0 and gradually drops from BA-2 to BA-4. Two genes known to be expressed in the ovary just prior and during sex change to male in protogynous species include folliculogenesis specific basic helix-loop-helix (factor in the germline alpha, *FIGLA*) and doublesex and mab-3 related transcription factor 1 (*DMRT1*)^47, 48^. While we expect the expression of these genes to transition in BA-4, neither *FIGLA* nor *DMRT1* are observed in salp blastozooids at any stage (BE-0 through BA-4). The only *DMRT* genes identified were *DMRT2* and *DMRTA2* which show low expression in all blastozooid stages. The role of *DMRT2* in sex differentiation is less understood but it is thought to play a role in spermatogenesis as they tend to be more highly expressed in the testis^66–68^.

The overlapping expression of genes involved in male and female gonadal development and functional gamete production immediately prior to blastozooid birth and throughout adult blastozooid stages do not fit the expected model of protogyny for salps (Figs. 1B, 5D). These data, taken with the recent observation of simultaneously occurring female and male physiology in an aggregate salp^69^ challenge our previous understanding of salp reproduction. Two alternative models are possible based on these data. (1) Salps may be reciprocal hermaphrodites which carry both a testis and ovary simultaneously and can therefore function as male or female. In this reproductive strategy, found in coral reef gobies (*Trimma spp.*)^70^, only one type of reproductive organ is mature and functional while the non-functional organ(s) is retained in an immature state, enabling rapid sex change based on specific environmental cues (such as body size, temperature, food abundance or population density). (2) Salps are synchronous (or true) hermaphrodites that carry both mature ovaries and testes. While the data herein supports synchronous mature gonads, a broader population-level assessment of salps are needed to differentiate among these models. Given that the Southern Ocean is under extreme annual and interannual fluctuations (e.g. temperature, salinity and food availability), the ability to release sperm and fertilize females when environmental conditions are optimal in austral spring allows for reproductive flexibility and the reliable production of oozoids to over-winter. The alternative models suggested by the gene expression profiles observed herein allow for a rapid reproductive response to rapidly changing environmental cues. Interestingly, while growth-related genes in blastozooids are markedly increasing in adulthood, these genes are most highly expressed in earlier embryonic stages in oozooids (Supplemental Fig. S5B,C, Supplemental Tables S30–S37). In clusters from all stage comparisons (Fig. 5A,B, Supplemental Figs. S5B, S6), ~ 50% of the genes were supported with full functional annotations by homology, indicating many may be novel, and potentially salp-specific, genes in growth and sexual development. Regardless, exploration of genes that were annotated revealed important unknown information about salp reproduction.

## Conclusion

Here we present the first complete genome assemblies for two sentinel species whose population dynamics are important indicators of changing oceanic conditions, the Southern Ocean salp, *S. thompsoni* and the North West Atlantic Ocean salp, *S. aspera*. Despite the ecological importance of salps in oceanic food web balance, these genomes are the only available for any salp species, providing a resource for understanding salp biology, tunicate genome evolution, and the factors that increase bloom formation, density and duration. These genomes presented significant technical challenges; *Salpa* genomes contain > 80% repetitive DNA content, an abundance of G4 motifs and a larger than average genome size for a tunicate species. The 12X genome size discrepancy among tunicate species has been attributed to differential repeat mobility^30^ and both *S. thompsoni and S. aspera* follow this trend, with larger genome sizes (742 Mb and 901 Mb, respectively) and higher repeat content (84% and 81%) than other tunicates (12–64%). Additionally, in this study we identified G4 motifs in predicted CREs of sex determination and cell proliferation genes suggesting they may be important regulators of gene expression in these processes. However, in-depth studies on where and when G4 structures are occurring are required to attribute their role in salp reproductive mechanisms.

The coincident timing of both male and female sexual development suggests blastozooid salp reproduction is more complicated than previously anticipated or described. It is known that protogynous species will initially develop gametes at sexual maturation with testicular tissue developing after sex change begins, resulting in a skewed ratio of female and male biased genes^47, 71^. However, in this study an overlap in the expression of male and female genes were observed as early as the blastozooid embryo (BE-4) and blastozooid adult (BA-0), earlier than expected for protogynous hermaphroditism. Evidence of genes involved in early and late spermatogenesis at stages before and during pregnancy suggests that gonad maturation and gamete production in the ovary and testis may occur at the onset of sexual maturation, consistent with simultaneous hermaphroditism^71^. Alternatively, the presence of gene expression profiles consistent with both male and female gonad maturation may indicate that sex change may occur rapidly and reciprocally, such that pregnant females may turn male before parturition. Both reproductive strategies allow for plasticity in generating male and female gametes rapidly, thus increasing the rate of fertilization during spring and summer, and consequently the number of oozoids available to overwinter and produce blastozooid chains the following year. However, details on the molecular pathways and the environmental cues required for hermaphroditism (synchronous, sequential and bidirectional) are lacking. In a lab environment, *Salpa fusiformis* developed testes despite not having an embryo (not fertilized), suggesting fertilization is not a prerequisite for male physiological development^72^. Salp-specific genes or hormones may be regulating this process; further analysis of gene expression from more individuals, an expanded developmental and environmental range, and histological analyses of gonads will help inform this study further. It is important to note that the classical model of protogynous hermaphroditism cannot be ruled out based on our data alone. In support of protogynous hermaphroditism, a recent report indicated sperm channels were only found in larger (i.e. older) blastozooids^73^.

As global ocean temperatures increase, and warmer seasons lengthen, the potential for bloom formation among salp species increases; increased production of oozoids from sexually producing salps in spring–summer coupled with increased survival of overwintering oozoids due to warming ocean temperatures will result in increased bloom frequency, density and biomass size. Concomitant with such increases in reproductive success are the detrimental impacts of salp to the trophic food web, carbon flux and marine-based human activities. The genetic toolkit presented herein confirms the importance of flexibility in growth and reproductive rates in order to adapt to varying environmental conditions and can better aid current predictive models and future impacts.

## Methods

### Sample collections

*Salpa thompsoni* samples were collected between January 9 and 29th, 2011 during a research cruise on the R/V *Polarstern* XXVII-2 in the Southern Ocean (Fig. 1A). All specimens were categorized by life stage (blastozooid versus oozooid; adult versus embryo), reproductive status (presence of gonads and embryos), and designation of maturational stages (0–5)^20^ (Fig. 1B). The gut was removed by dissection to avoid DNA contamination from prey before muscular tissue was flash-frozen with liquid nitrogen and stored at −80 °C.

*S. aspera* samples were collected on the R/V *Neil Armstrong* research cruise (SVC-VI) in the Atlantic Ocean between June 17 and 23, 2016 (Fig. 1A). Samples were collected in a tow net at a depth of 0–20 m. Whole samples were collected in RNA later and stored at −80 °C. Immediately prior to extraction, the gut and attached zooplankton were removed and the muscle tissue was separated and rinsed in fresh sterile sea water for DNA extraction.

### DNA extraction and genome sequencing of *S. thompsoni* and *S. aspera*

High molecular weight (HMW) DNA was extracted from the muscle tissue of an oozooid *S. thompsoni* using a basic phenol:chloroform extraction following standard procedures for long-read sequencing on ONT platforms. Prior to library preparation, the DNA was sheared to 20 kb using the Covaris g-TUBE. Four libraries were prepared for ONT sequencing using the 1D Genomic DNA by ligation library preparation kit (SQK-LSK109). Three libraries were run on the PromethION (PRO-002) and one on the MinION (MIN-107). The fifth library was prepared using the WGA preparation; HMW DNA (300 pg) was amplified with the Qiagen Repli-G kit protocol as per the manufacturer’s instructions with an overnight incubation at 30 °C and run on the PromethION (PRO-002). For all libraries, 25–30 femtomoles of the library were initially loaded for sequencing and after ~ 24 h the flow cells were flushed, DNased and loaded with an additional 25–30 femtomoles. In total, 24,031,332 reads with passing quality (≥ Q7) were generated with an average N50 length of 9.9 kb, totaling 129.34 Gb (Supplemental Table S1).

HMW DNA from the muscle tissue of *S. aspera* was extracted using the Qiagen G-tip 100/g kit. The same protocol for sequencing as described above for *S. thompsoni* was utilized for *S. aspera*. Two libraries were run on the PromethION (PRO-002) and one on the MinION (MIN-107). The fourth library was prepared using WGA; HMW DNA (300 pg) was amplified with the Qiagen Repli-G kit protocol as above. In total, 9,361,641 reads with passing quality (≥ Q7) were generated with an average N50 length of 17.5 kb, totaling 78 Gb (Supplemental Table S1).

For all samples, reads were base-called during sequencing using the Guppy high accuracy base-caller version 2.1.3 (ONT). Once sequencing was complete, quality statistics were calculated using *Nanoplot* version 1.21.0 to assess the number of reads, read length and read quality^74^.

### Assembly of the *S. thompsoni* genome

Passing reads (≥ Q7) from all runs were combined and assembled using a variety of methods to produce the highest scoring *S. thompsoni* genome assembly based on standard quality metrics (Supplemental Table S1). Quality and completeness of assemblies were measured at each step and for each assembler using *QUAST*^75^ and *BUSCO* v5 with OrthoDB v10 database^23^ to guide the assembly process (Supplemental Table S2). First, reads were assembled using two long-read assemblers, *Flye* v2.4.2^25^ and *Shasta* v0.7.0^24^*,* to identify the best performing assembler for salp sequences. Input genome size used for *Flye* was 600 Mb based on a genome size estimate from Jue et al^22^. For *Shasta*, no input genome size was required, and a minimum read length of 500 bp was used. Contigs less than 3 kb in length were removed from each assembly. *Shasta* produced the highest quality assembly (Supplemental Table S2). Next, the *Shasta* assembly was polished to remove errors implicit to ONT sequencing with both *Nanopolish* v0.11.1^76^ and *Medaka* v1.3.2 (ONT) and to identify the best polishing algorithm; *Medaka* v1.3.2 was selected as the best polisher for this assembly (Supplemental Table S2). Once polished, haplotigs were purged from both assemblies using *Purge Haplotigs*^77^. *Purge Haplotigs* cutoffs were manually input based on the coverage histogram from step 1 of the *Purge Haplotigs* pipeline (*Nanopolish* assembly cutoffs were: -l 15 -m 43 -h 90; *Shasta* assembly cutoffs: -l 12 -m 43 -h 90). The *Shasta* assembled and *Medaka* polished genome was selected as the final assembly based on *QUAST* and *BUSCO* statistics. Contamination in the final genome was identified and removed using *Centrifuge* v1.0.4-beta^78^. For contamination identification, a database containing all complete archaeal, bacterial, viral and human sequences was used with a minimum hit cutoff of 100 bp. Additionally, to ensure no major misassembles occurred during the assembly, the raw reads were mapped back to the genome using *Minimap2* v2.15^79, 80^ (Supplemental Table S3). The Darwin Tree of Life naming convention was adapted for the *S. thompsoni* assembly including a one letter prefix representing the class (t = Thaliacea), a six letter combination consisting of the first three letters of the genus/species (SalTho), the individual used for this assembly (2nd individual used) and the version of the genome made from the specimen (version 1): *tSalTho2.1* (GRIT/darwin-tree-of-life-sample-naming GitLab).

### Genome assembly of the *S. aspera* genome

Passing reads (≥ Q7) from all runs were combined and assembled to create the *S. aspera* genome (Supplemental Table S1). Quality and completeness of assemblies were measured at each step using *QUAST* and *BUSCO* v5 with OrthoDB v10 database to guide the assembly process (Supplemental Table S5). Reads were assembled, polished, and further processed as above to identify the best performing assembler, with the same input genome size (600 Mb) used for *S. thompsoni*. The best quality *S. aspera* assembly utilized *Flye* for assembly. The *S. aspera* assembly is named based on the Darwin tree of life naming convention: *tSalAsp1.1* (GRIT / darwin-tree-of-life-sample-naming · GitLab).

### Repeat annotations

Repeats were identified using *RepeatModeler*^81^ alone and with a combined method using *RepeatModeler* v2.01, *Transposon PSI* v1.0.0^82^, and *LTRharvest* v1.5.10^83^ to create de novo repeat libraries from the *S. thompsoni* and *S.* *aspera* genomes. For the combined library, each program was run individually before being compiled into one library. The combined de novo library was clustered using *Usearch* v9.0.2132^84^ with a minimum match of 80% to remove redundant sequences that may have been identified in multiple programs. The genomes were then soft masked using the *RepeatModeler* only library and the combined de novo library with *RepeatMasker* v4.0.9^85^. Repeat statistics were compared for each library to determine the best repeat annotation. The genome masked with the combined de novo repeat library was used for downstream analyses (Supplemental Table S10). *RepeatMasker* was also re-run by the authors on *B. schlosseri, C. intestinalis* and *O. dioica* with a de novo library from *RepeatModeler* (Supplemental Table S11).

### Gene annotations

Once the *S. thompsoni* genome was soft-masked for repeats, it was annotated using *Braker* v2.1.4^37–41^. *S. thompsoni* RNA-seq reads and proteins from the developmental transcriptome were used to aid gene predictions. Proteins were aligned within the *Braker* pipeline using *GeneMark-ET* v4.59^41^ and *Augustus* v3.2.3. Gene models were checked using *BUSCO* for completeness and *gFACS*^86^ for overall statistics. Gene predictions were then filtered to remove erroneous models using *gFACS* with varying parameters to identify the best gene model set (Supplemental Fig. S4, Supplemental Table S12). Final filtering parameters allowed: (1) all incomplete genes to be retained in the gene set; (2) genes with minimum intron and exon size as small as 6 bp; (3) genes with a minimum CDS size to be as small as 16 bp; and (4) collapse overlapping genes allowing only unique genes to be kept. Mono-exonic gene models were separated and filtered with *InterProScan* v5.35-74.0^87^ against the Pfam database. Mono-exonics with no protein match were removed using an in-house python script and mono-exonics with protein matches were kept in the final annotations. Final gene models were annotated with *EnTAP*^27^.

### *S. thompsoni* RNA extractions

Twenty-one samples were chosen from a range of developmental stages and based on the availability of samples (Supplemental Table S6). The embryonic stages were removed from adult tissue during dissection, ensuring that embryo and parent pairs were used for sequencing. Samples include three oozooid adults stage 4 (OA-4), and their young blastozooids (BE-4), three stage 0 blastozooid adults (BA-0) which had no embryos, three stage 2 blastozooid adults (BA-2) and their oozooid embryos (OE-2) and three stage 4 blastozooid adults (BA-4) and their oozooid embryos (OE-4) (Supplemental Fig. S2A, Supplemental Table S6).

RNA was extracted from adult muscle and embryonic frozen tissue separately (TRIzol LS/Invitrogen). All samples were shaken in the MPBio benchtop homogenizer at 4 M/S for 20 s. After each program, samples were put on ice and visually checked to confirm tissue dissociation. If tissue was remaining, shaking was repeated until samples were homogenized, placing tubes on ice after each 20 s interval. Once homogenized, samples were carried through the miRVana miRNA isolation protocol (Invitrogen) starting at the Organic Extraction Section of the manufacturer's protocol. Both large (> 200 bp) and small (< 200 bp) fractions of RNA from each sample were kept for mRNA and smRNA sequencing.

### Transcriptome sequencing and assembly for *S. thompsoni*

The large fraction of total RNA for each sample was prepared for sequencing using the TruSeq Stranded mRNA Preparation Guide according to the manufacturer’s instructions (Illumina). Paired-end 150 bp sequencing was performed using the Illumina HiSeq. All samples were individually quality checked using *FastQC* v0.11.7^88^ before removing adapters with *cutadapt* v1.14^89^. Reads less than 50 bp in length and a quality score < 30 were removed using *Sickle* v1.33^90^. Read quality was assessed again after trimming and quality filtering using *FastQC* v0.11.7. Additionally, raw RNA reads from each sample were mapped back to the genome to check the quality and specificity of reads using *Hisat* v2.1.0^91^ (Supplemental Table S8). All samples were included in the transcriptome assembly.

All samples were assembled individually using *Trinity* v2.8.5^92, 93^. The individual assemblies were combined and redundant transcripts were removed using *vsearch*^94^ (minimum ID cutoff: 90%). Coding regions were predicted using *Transdecoder* v5.3.0^95^ and all sequences less than 300 bp in length were removed. Statistics and completeness were assessed using *QUAST* and *BUSCO* v5 with the metazoa orthoDB v10 database. The final transcriptome was annotated using *EnTAP* utilizing the NCBI RefSeq and a Non-redundant protein sequence database with entries from GenPept, Swissprot, PIR, PDF, and PDB databases.

### Differential expression

Trimmed and quality filtered RNA reads from each sample were individually mapped to the developmental transcriptome and counted using *kallisto* v0.44.0^96^. Differential expression, clustering analysis and visualization was completed using *TCseq*^97^ and *EdgeR’s* generalized linear model (GLM) method^98^ in R. Significant differential events were categorized as having a log2-fold change between 2 and -2 with an adjusted p-value less than 0.05. Regions falling into these criteria were then used in temporal pattern analysis with *TCseq* using log fold change values and normalized using RPKM. The Calinski-Harabasz index was used to test for the appropriate number of clusters for the analysis using the *vegan* package in R^99^. Once clusters were generated, *EnTAP* annotations from the transcriptome were used to determine gene identity and GO enrichment was performed for each cluster using *GoSeq* in R^100^.

### *S. thompsoni* small RNA sequencing and differential expression

The small RNA fraction for each sample was prepared for sequencing using the TruSeq smRNA Preparation Guide (Illumina). Size selection was performed using the Pippin Prep (Sage Science) with a dye-free 3% for ~ 1 h and 20 min to collect sizes between 135 and 200 bp. Size selected samples were purified using the Qiagen PCR Purification kit (Qiagen) following the manufacturer's protocol. Final libraries were pooled and sequenced on the Illumina NextSeq v2.5.

Reads were filtered to remove the 3ʹ smRNA adapter using *fastx_clipper*^101^ and by quality (> 30) and sequence length (< 15 bp) with *sickle* v1.33^90^. Quality was assessed after each step using *FastQC* v0.11.7. In addition, cleaned and trimmed reads were mapped back to the *S. thompsoni* genome to check quality using *bowtie* v1.1.2 and default settings (allowing 2 mismatches)^102^. Known and novel miRNAs were classified using miRDeep2 v2.0.0.8^103^. Before analysis, low complexity sequences were removed using the Usearch -filter lowc command with default parameters^94^ before mapping to the *S. thompsoni* genome using the *miRDeep* mapper script (default settings and the -m flag to collapse reads). The mapped miRNAs were then classified against the miRBase database containing all available sequences^104^. All miRNA sequences were then quantified using the miRDeep quantifier script before inputting into *DESeq*2 v1.30.1 for differential expression analyses^103, 105^. With *DESeq2*, shrunken log fold change was used to account for variance of lowly expressed miRNAs^105^. Reads were normalized using variance stabilization before visualization of the top 30 DE miRNAs between stages^105^.

### Differential expression of *S. thompsoni* miRNAs

Differential expression of miRNAs between developmental stages was analyzed using *miRDeep2* v2.0.0.8^103^. Before analysis, low complexity sequences in the miRNA reads pool (18-24nt) were removed using *Usearch* (options: -filter lowc) with default parameters on all samples combined. The *miRDeep* mapper script was used to first map the reads to the *S. thompsoni* genome with default settings and then collapse reads (option: -m). The *miRDeep* script was run using a miRbase database containing all available sequences. The sequences mapped to the database were quantified using the *miRDeep* quantifier script to input into *DESeq2*^105^ for differential expression analyses. Shrunken log fold change was used to account for variance of lowly expressed miRNAs. Reads were normalized using both rlog transformation and variance stabilization. Variance stabilized data was used to visualize the top 30 differentially expressed miRNAs between stages.

### G-quadruplex identification

Species chosen for comparison were downloaded from NCBI and genome statistics were checked using *QUAST* version 5.0.2 before running *G4hunter* (Supplemental Table S9)^28^. G-quadruplex (G4) motifs were identified in all genomes with *G4hunter* using default parameters (window size: 25 and score threshold: 1.4). The total number of G4 sequences predicted, the number of G4s predicted per contig, and summary statistics were counted using in-house python scripts. The *G4hunter* output was converted to a bed file using an in-house python script for use with *Bedtools* v2.29.0^106^. *Bedtools* intersect with parameters allowing all overlaps and complete overlaps only were used to identify G4 overlaps with genes. The percentage of G4’s that overlap genes, introns and exons were calculated.

To identify if G4’s could be transcriptional regulators or associated with cis-regulatory elements (CREs), G4 motifs in potential promoters (500 bp to 1 kb) and enhancers (5 kb) up and down stream of genes were analyzed using *Bedtools* flank. While enhancers can be up to 1 Mb away, the analysis was capped at 5 kb due to the fragmentation of the genome and the observation that CREs are within 2–4 kb of the transcriptional start site in tunicates^107^. *Bedtools* overlap was then used with the same parameters as above to identify regions with G4’s within the 5 kb regions. The total amount of G4 motifs predicted divided by the genome assembly size was used to calculate expected distribution of G4 sequences if distributed equally across the genome. Distribution was calculated by counting the number of predicted G4 motifs per 10 kb window of the genome using *Bedtools* map.

### Supplementary Information

Supplementary Figures.

Supplementary Tables.

## Data Availability

All sequencing data have been deposited to NCBI’s SRA database and GenBank for *S. thompsoni* under the project accession number PRJNA783907 and for *S. aspera* under the project accession number PRJNA783906. The genome assemblies, repeat annotations, gene annotations, G4 motif predictions and transcriptome assembly are also available in Zenodo (10.5281/zenodo.7435265).

## Code availability

All code used in this study is publicly available and can be accessed via Gitlab (*Salpa thompsoni*: https://gitlab.com/kcastellano13/salpa_thompsoni_genome, *Salpa aspera:* https://gitlab.com/kcastellano13/Salpa_aspera_Genome).
