# Supplementary material for: Salpa genome and developmental transcriptome analyses reveal molecular flexibility enabling reproductive success in a rapidly changing environment
Source: Sci Rep. 2023 Nov 29;13:21056. doi: 10.1038/s41598-023-47429-6 (PMC10686999; doi:10.1038/s41598-023-47429-6)

Castellano et al_Supp Figure 1

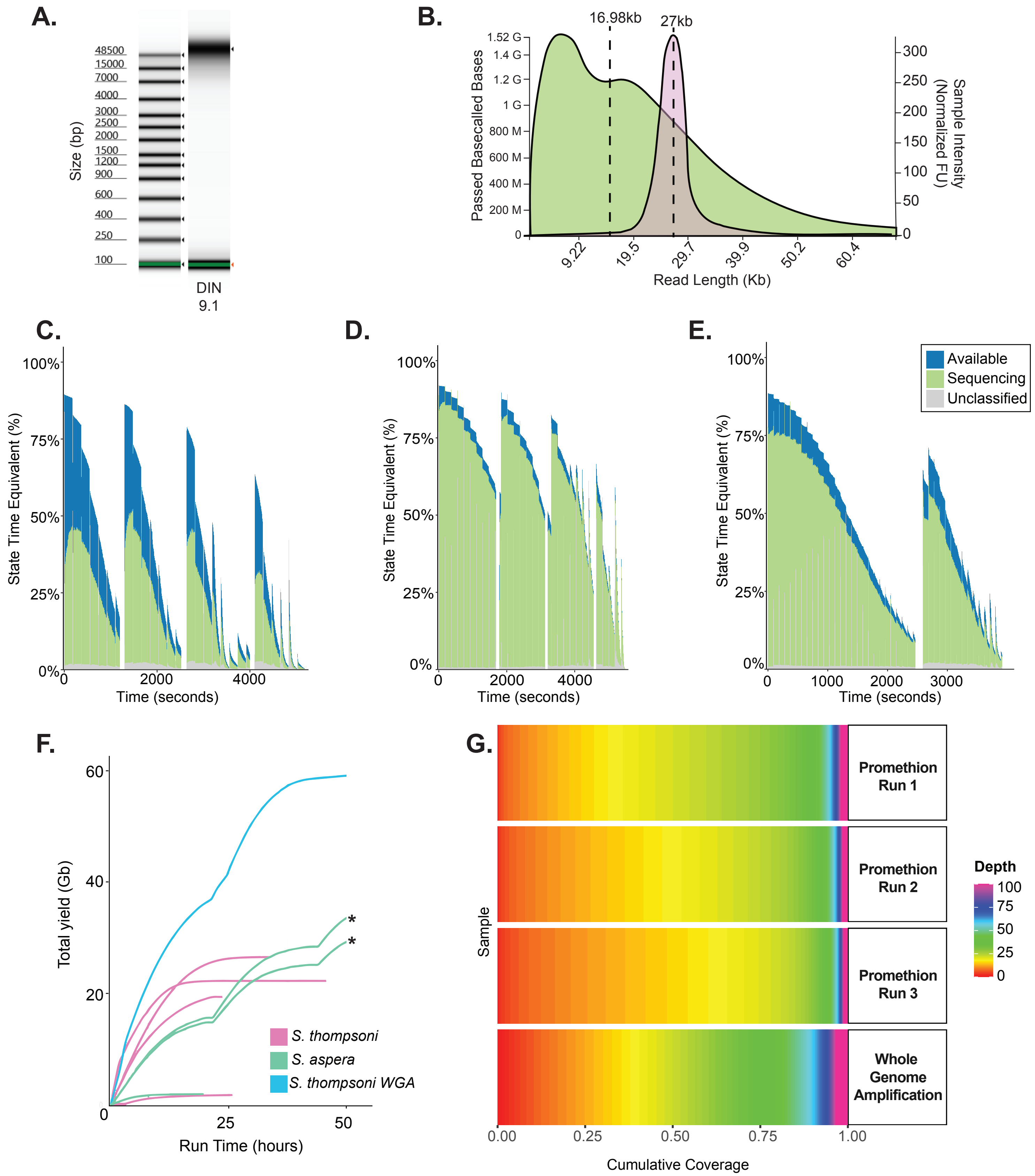

Castellano et al_Supp Figure 2

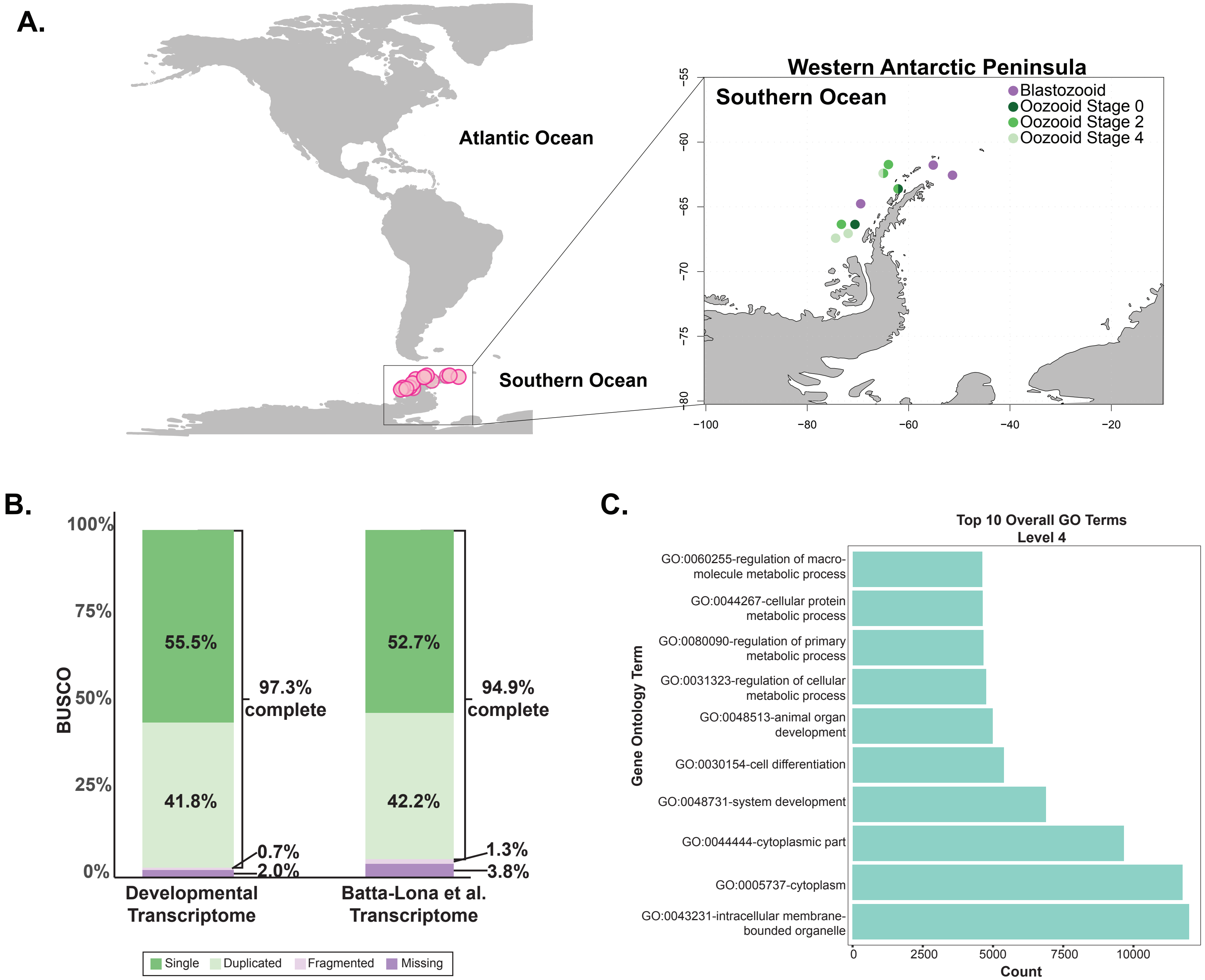

Castellano et al_Supp Figure 3

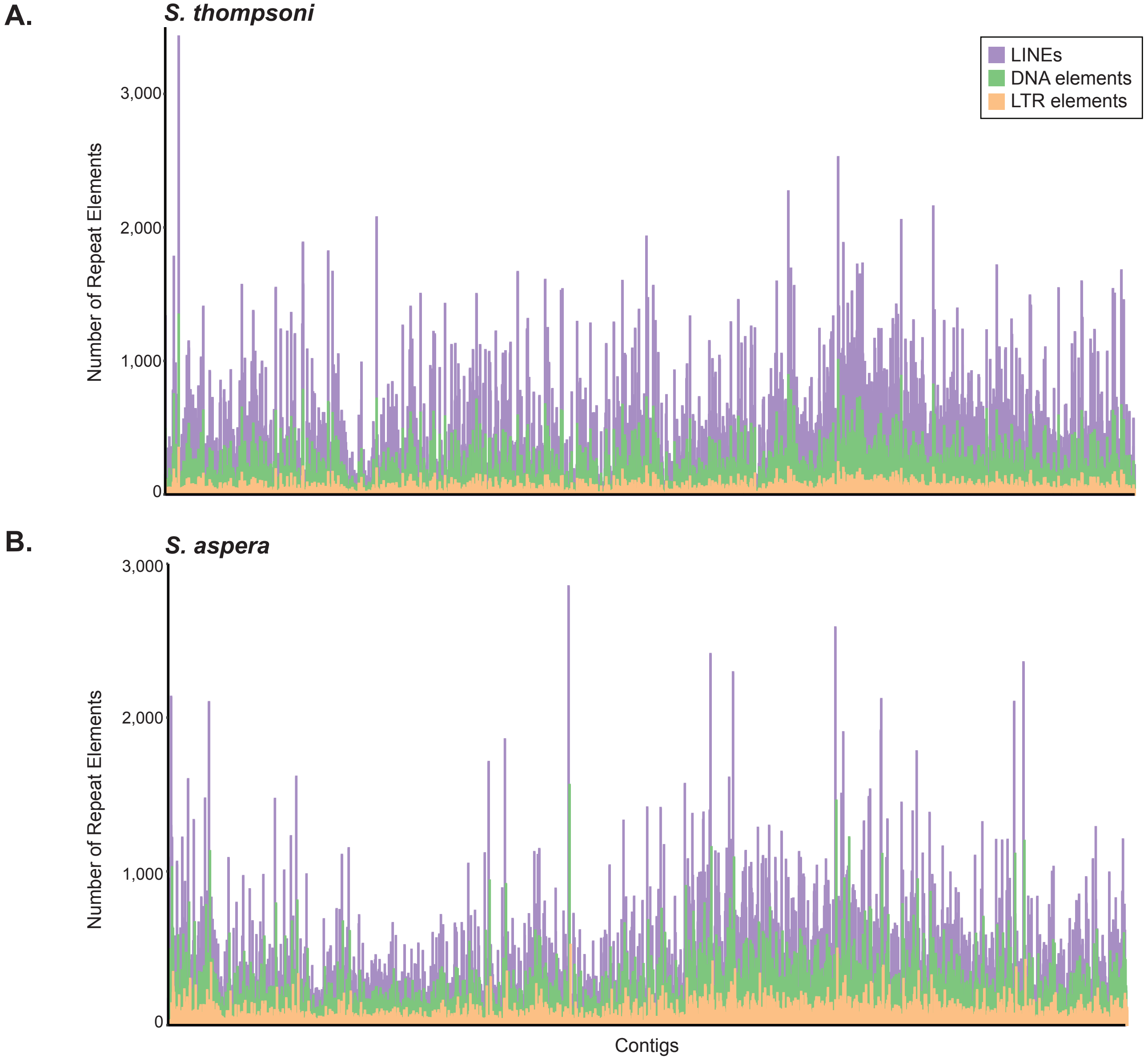

Castellano et al_Supp Figure 4

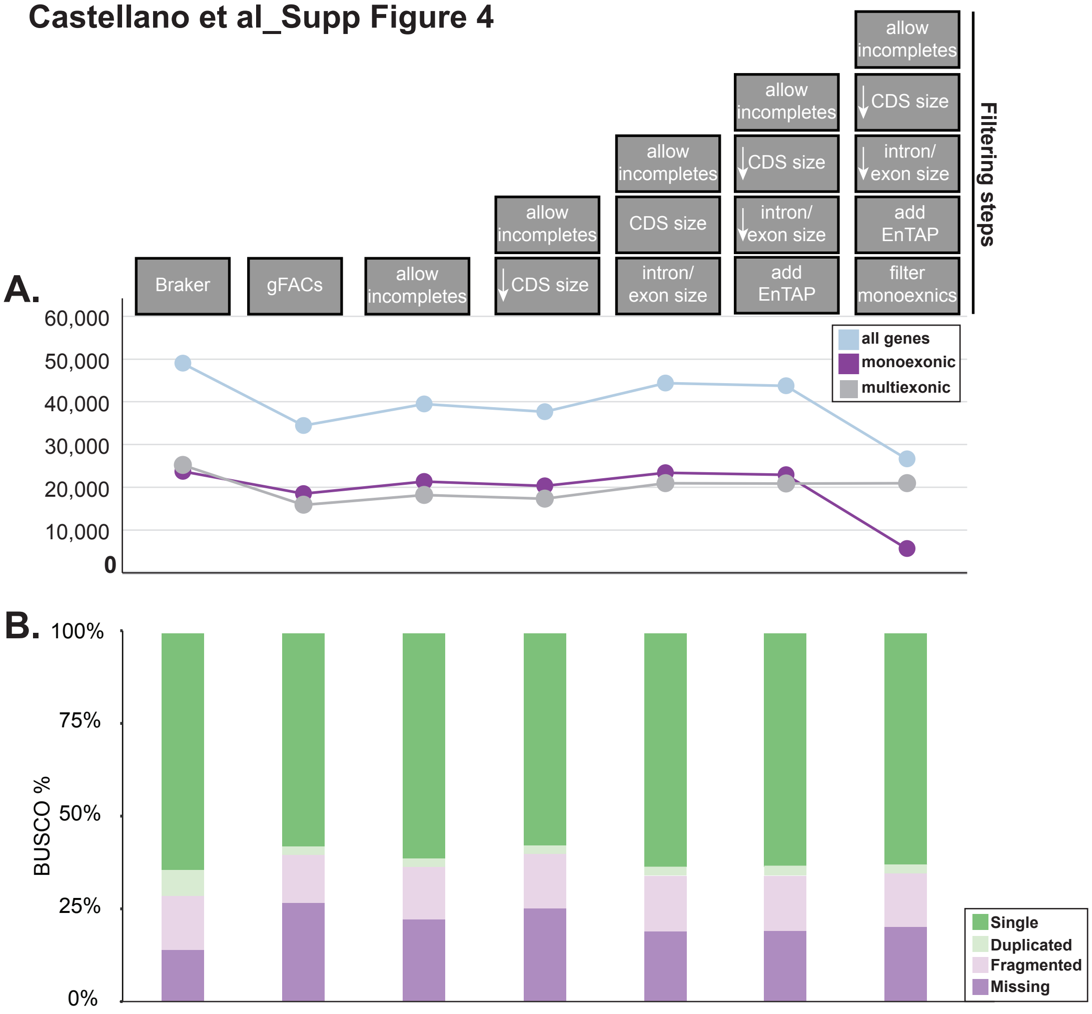

Castellano et al_Supplemental Figure 5

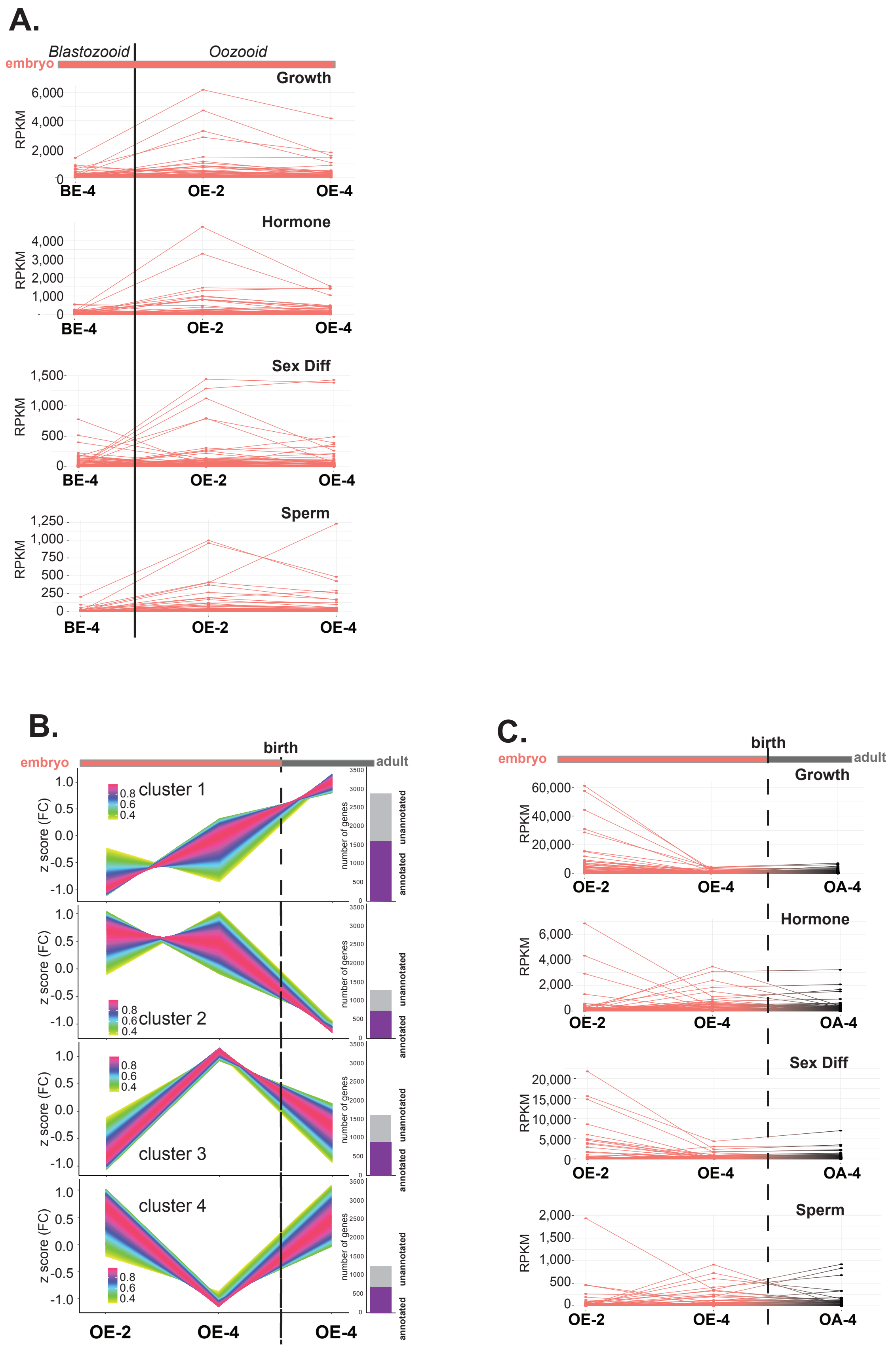

Castellano et al_Supplemental Figure 6

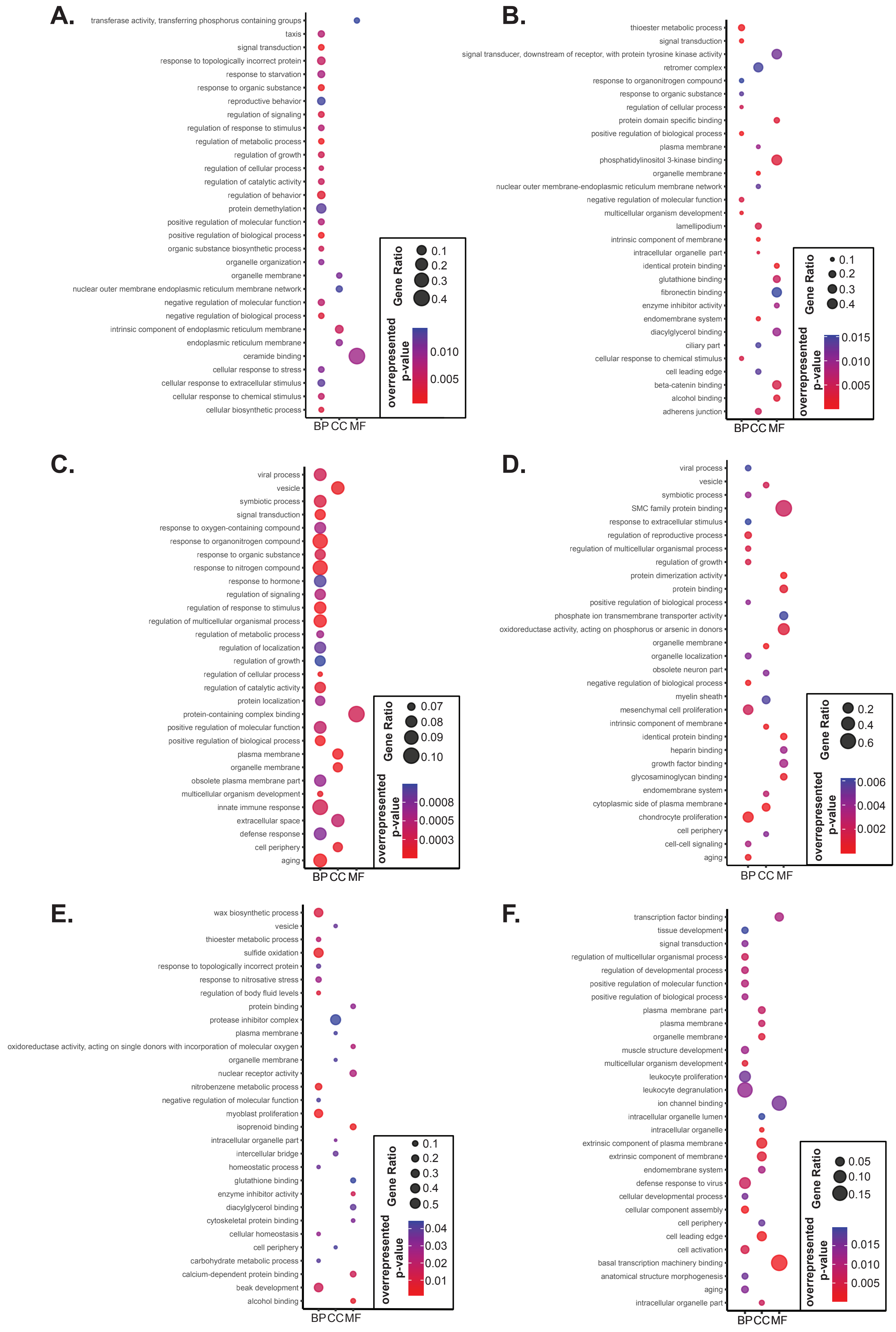

Castellano et al_Supplemental Figure 6 (Continued)

G.

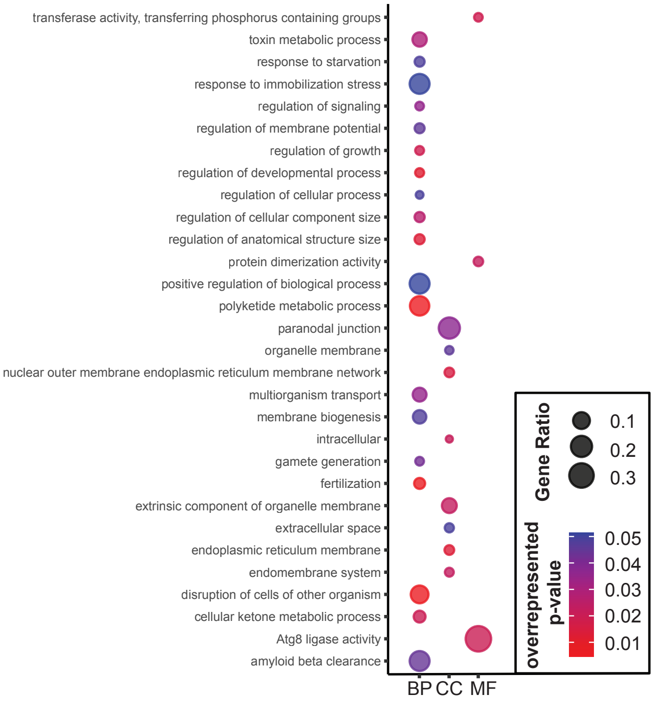

H.

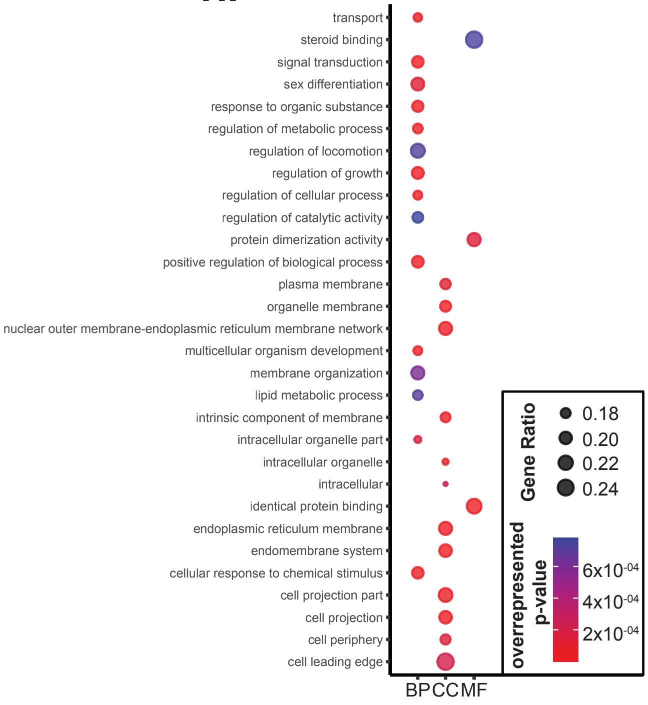

I.

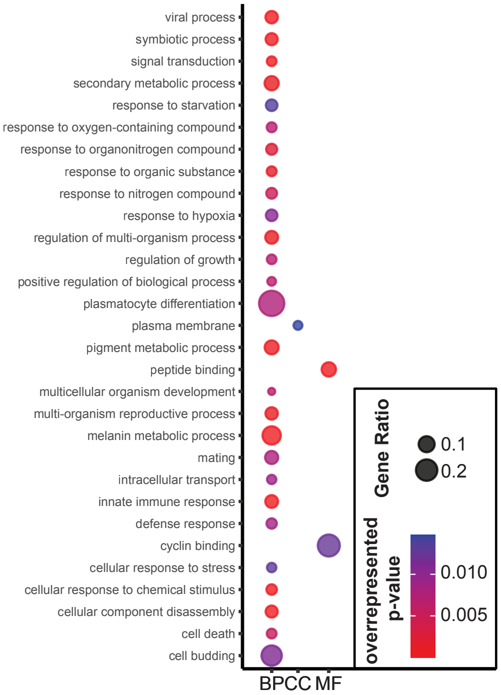

J.

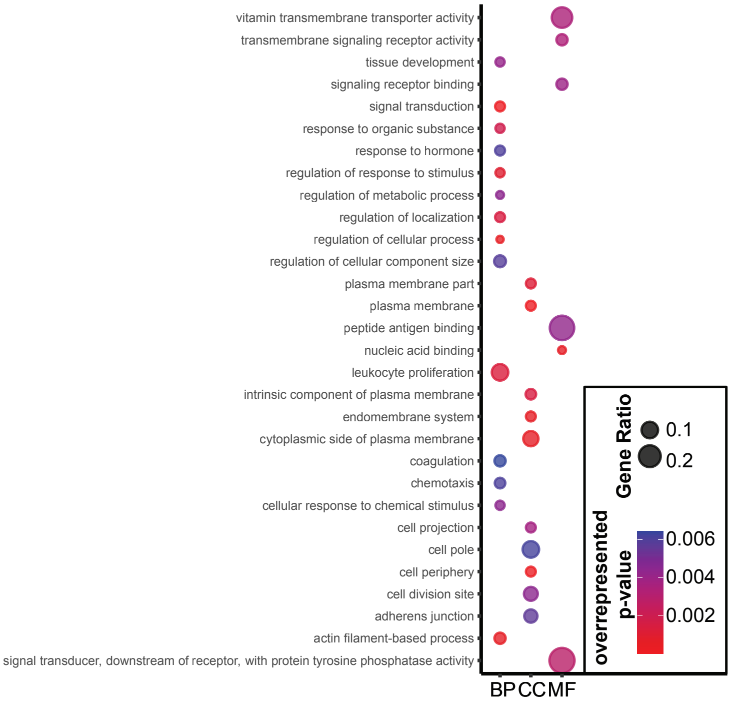

Supplement: Supplementary file 1 — Supplementary Figures. [file 41598_2023_47429_MOESM1_ESM.pdf]
